# Curcumin Inhibits Non-Small Cell Lung Cancer Cells Metastasis through the Adiponectin/NF-κb/MMPs Signaling Pathway

**DOI:** 10.1371/journal.pone.0144462

**Published:** 2015-12-10

**Authors:** Jong-Rung Tsai, Po-Len Liu, Yung-Hsiang Chen, Shah-Hwa Chou, Yu-Jen Cheng, Jhi-Jhu Hwang, Inn-Wen Chong

**Affiliations:** 1 Department of Respiratory Therapy, College of Medicine, Kaohsiung Medical University, Kaohsiung, Taiwan; 2 Department of Internal Medicine, Kaohsiung Medical University Hospital, Kaohsiung Medical University, Kaohsiung, Taiwan; 3 Graduate Institute of Integrated Medicine, China Medical University, Taichung, Taiwan; 4 Department of Surgery, Division of Chest Surgery, Kaohsiung Medical University Hospital, Kaohsiung Medical University, Kaohsiung, Taiwan; 5 Department of Health Management, Division of Thoracic Surgery, Department of Surgery, E-Da Hospital, I-Shou University, Kaohsiung, Taiwan; Institute of Biomedical Sciences, TAIWAN

## Abstract

Adipose tissue is now considered as an endocrine organ involved in metabolic and inflammatory reactions. Adiponectin, a 244–amino acid peptide hormone, is associated with insulin resistance and carcinogenesis. Curcumin (diferuloylmethane) is the principal curcuminoid of the popular Indian spice, turmeric. Curcumin possesses antitumor effects, including the inhibition of neovascularization and regulation of cell cycle and apoptosis. However, the effects of adiponectin and curcumin on non-small cell lung cancer (NSCLC) remain unclear. In this study, we evaluated the expression of adiponectin in paired tumors and normal lung tissues from 77 patients with NSCLC using real-time polymerase chain reaction, western blotting, and immunohistochemistry. Kaplan–Meier survival analysis showed that patients with low adiponectin expression ratio (<1) had significantly longer survival time than those with high expression ratio (>1) (*p* = 0.015). Curcumin inhibited the migratory and invasive ability of A549 cells via the inhibition of adiponectin expression by blocking the adiponectin receptor 1. Curcumin treatment also inhibited the *in vivo* tumor growth of A549 cells and adiponectin expression. These results suggest that adiponectin can be a prognostic indicator of NSCLC. The effect of curcumin in decreasing the migratory and invasive ability of A549 cells by inhibiting adiponectin expression is probably mediated through NF-κB/MMP pathways. Curcumin could be an important potential adjuvant therapeutic agent for lung cancer in the future.

## Introduction

Lung cancer remains the leading cause of cancer-related mortality worldwide and in Taiwan. Despite great advances in the understanding of lung carcinogenesis and in novel treatments in the past few decades, the overall 5-year survival rate remains poor. Innovative research effort must be redirected to investigate potential markers of prognosis, mechanisms of lung carcinogenesis, and adjuvant therapy.

Adipose tissue is presently considered as an endocrine organ that secretes several cytokines (adipokines) [1], including adiponectin and leptin. Adiponectin is a 244–amino acid polypeptide that modulates numerous metabolic processes such as glucose regulation and fatty acid catabolism [2]. It exerts significant effects on metabolism and lipogenesis, as well as on the regulation of human inflammatory responses [3]. In adults, adiponectin concentrations are inversely correlated with body fat percentage and insulin resistance [4]. Adiponectin has antidiabetic, anti-atherogenic, anti-inflammatory, and anti-angiogenic properties [5].

The role of adiponectin in carcinogenesis is controversial [5]. In obesity-associated malignancies such as endometrial cancer, post-menopausal breast cancer, colon cancer, renal cancer, and hematologic malignancies, adiponectin expression is positively correlated with the risk of malignancy. Furthermore, low adiponectin concentrations have been reported in gastric and prostate cancer [6]. However, in non-obesity-associated cancers, such as lung cancer, serum adiponectin is not a major predictor of risk [7].

Adiponectin receptors 1 and 2 act directly on tumor cells by binding and activating adiponectin receptors and downstream signaling pathways [5]. Adiponectin possesses anti-angiogenesis and antitumor ability, which is effected through caspase-mediated endothelial cell apoptosis [8]. It can also inhibit liver tumor growth and metastasis by suppressing tumor angiogenesis and downregulating the ROCK/IP10/matrix metalloproteinase (MMP) -9 pathway [9].

However, adiponectin is a potential marker of prostate cancer progression [10]. In chondrosarcoma, it mediates the migration of human chondrosarcoma cells by the transcriptional upregulation of alpha2beta1 integrin and activation of AdipoR receptor, AMPK, p38, and NF-κB pathways [11]. Nevertheless, its role in lung cancer remains unclear [12,13]. Petridou et al. reported that circulating adiponectin levels are not correlated with lung cancer stages [14]. The expression of adiponectin receptor 1 is a favorable prognostic factor for lung cancer [15].

Curcumin (diferuloylmethane), a natural compound extracted from *Curcuma longa*, has been used since ancient times in the Orient as a healing agent for various illnesses. Curcumin has several pharmacological activities, including anti-inflammatory [16], antioxidant [16], antimicrobial [17], and anticancer effects [18–21]. Its anticancer effects are manifested during cell proliferation, invasion, metastasis, apoptosis, and angiogenesis [22]. For example, curcumin can induce cellular apoptosis in non-small cell lung cancer (NSCLC) [23,24]. The molecular basis of its anticarcinogenic effect is attributed to its effect on several targets, including transcription factors, growth regulators, adhesion molecules, apoptotic genes, angiogenesis regulators, and cellular signaling molecules [25]. Although curcumin inhibits lung cancer cell migration and invasion via Rac-1 or Wnt/B-catenin pathways, the regulatory mechanism of curcumin in lung cancer remains unknown.

Most lung cancers are diagnosed at an advanced stage when treatment is relatively ineffective. How to augment currently available chemotherapeutic treatments with adjunctive herbal medicines, which may decrease side effects and toxicity without compromising therapeutic efficacy, has become a popular approach in recent decades. Curcumin is one such potential candidate. This study explored the diagnostic and prognostic roles of adiponectin in NSCLC. Using *in vitro* A549 cell culture and an animal model, we demonstrated the potential role of curcumin for treating lung cancer. The exact molecular mechanism of curcumin in mediating adiponectin effect was also investigated. Consequently, the potential of curcumin as an adjuvant agent in lung cancer treatment will also be explored.

## Results

### Demographic data and adiponectin expression in NSCLC patients

**Fig 1:**
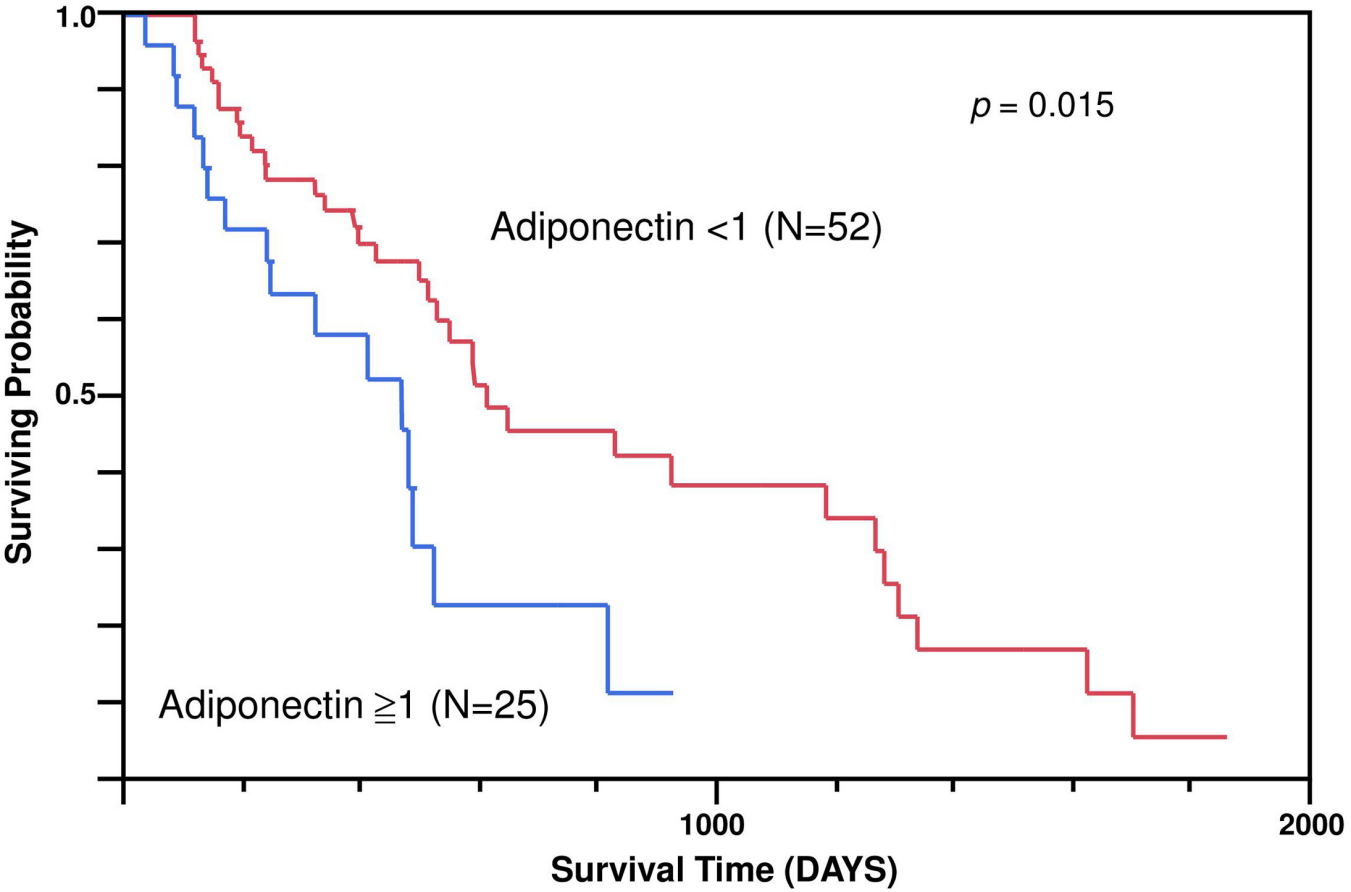
Survival curve of non-small cell lung cancer patients with high and low expression of adiponectin assessed using Kaplan–Meier analysis.

Of the 77 NSCLC patients in this study, 58 (75%) had histologically confirmed adenocarcinoma and 19 (25%) had squamous cell carcinoma. Their average age was 61.6 ± 10.3 years (range, 36–78 years). Adiponectin expression was not correlated with tumor (T), lymph nodes (N), and stages. NSCLC patients with metastasis had significantly higher adiponectin expression ratio (Table 1). The Kaplan–Meier survival analysis showed that NSCLC patients with a low adiponectin expression ratio had a significantly longer survival time than those with a high adiponectin expression ratio (*p* = 0.015) (Fig 1). Multivariate-adjusted risk ratios were computed from Cox regression with the additional variables of sex (male vs. female), metastasis, tumor, lymph node involvement, and stages (Table 2). The high adiponectin expression group had a 2.40-fold higher mortality risk (*p* = 0.04) than the low expression group.

**Table 1:** Relationship between adiponectin expression ratio and demographic characteristics of non-small cell lung cancer (NSCLC) patients.

	Number	Mean±SD	*P*
**STAGE**			0.28
1 + 2	37	0.90±1.07	
3 + 4	40	1.21±1.40	
**Tumor (T)**			0.35
1	19	1.32±2.01	
2	39	0.96±0.83	
3	6	0.38±0.3	
4	13	1.3±1.12	
**Node (N)**			0.24
0	42	1.32±1.57	
1	15	0.63±0.36	
2	14	0.81±0.8	
3	6	0.8±0.57	
**Metastasis (M)**			0.01*
0	53	0.83±0.97	
1	24	1.63±1.64	
**Pathology**			0.06
SQ	19	1.72±2.28	
AD	58	0.96±1.2	
**Sex**			0.25
Male	47	1.20±1.4	
Female	30	0.85±0.98	
**Smoking**			0.07
yes	35	1.35±1.51	
no	42	0.88±0.7	

SQ: squamous cell carcinoma, AD: adenocarcinoma (*p < 0.05)

**Table 2:** Multivariate cox regression analysis of mortality.

Term		Risk Ratio	Lower 95%	Upper 95%	*p*
Adiponectin expression > 1		2.40	1.05	5.44	0.04*
T	2 vs. 1	1.69	0.75	4.06	0.21
	3 vs. 1	2.99	0.73	12.22	0.13
	4 vs. 1	2.48	0.83	7.63	0.10
N	1 vs. 0	1.99	0.81	4.78	0.13
	2 vs. 0	2.18	0.68	6.72	0.19
	3 vs. 0	2.46	0.62	8.79	0.19
Metastasis		1.83	0.60	5.55	0.29
Pathology	AD vs. SQ	1.08	0.53	2.33	0.84
Smoking		1.87	0.92	3.78	0.09
Stage	(3 + 4) vs. (1 + 2)	0.49	2.10	2.03	0.12

*p < 0.05

### Expression levels of adiponectin, adiponectin receptors, and MMPs in NSCLC patients

**Fig 2:**
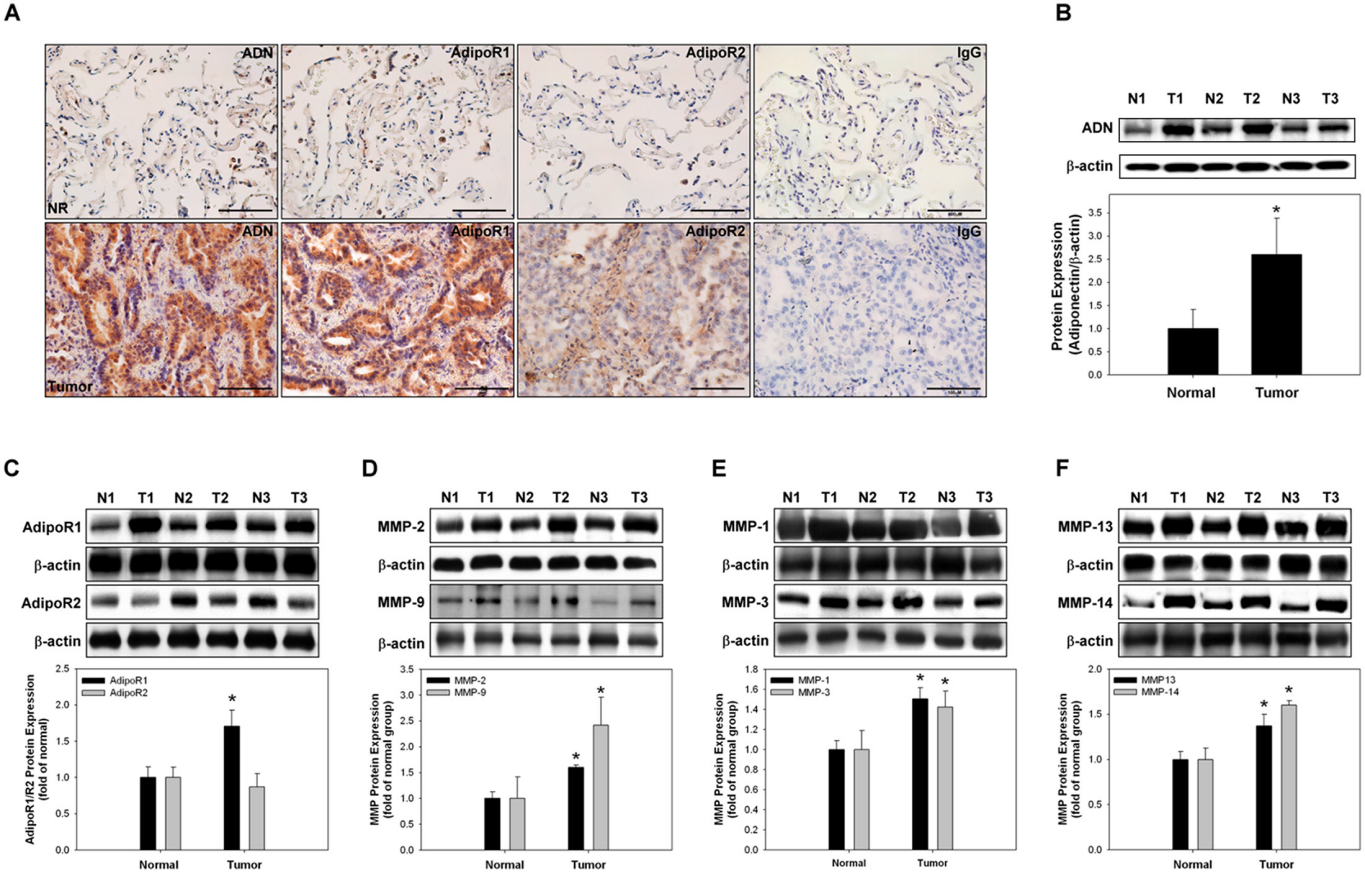
Expression levels of adiponectin, adiponectin receptor 1, adiponectin receptor 2, and matrix metalloproteinases (MMPs) in non-small cell lung cancer tissues. **(A)** Samples (lung cancer and the corresponding normal adjacent lung tissues) were analyzed with antibodies to adiponectin, adiponectin receptor 1, and adiponectin receptor 2 by immunohistochemical staining (DAB staining and hematoxylin counterstaining). For negative controls, the antibody was replaced with control IgG. **(B-C)** Expression levels of adiponectin, adiponectin receptor 1, and adiponectin receptor 2 in three pairs of lung cancer and normal tissues analyzed by western blotting. **(D–F)** Gelatin zymography was used to analyze the activities of MMP-2/MMP-9, MMP-1/MMP-3, and MMP-13/MMP-14. *p < 0.05 vs. normal lung tissue, with representative data from three different patients with NSCLC. T, tumor; N, normal; ADN, Adiponectin; AdipoR1, adiponectin receptor 1; AdipoR2, adiponectin receptor 2.

The expression levels of adiponectin, adiponectin receptors, and MMPs of NSCLC patients were analyzed (Fig 2). Immunohistochemical staining and western blotting of lung cancer tissue showed an increased expression of adiponectin and adiponectin receptor 1 in the lung cancer tissue than in normal lung tissue (Fig 2A–2C). All MMPs were also increased in the lung cancer tissue than in the normal lung tissue (Fig 2D–2F).

### Cytotoxic effect of curcumin on the expression of adiponectin and adiponectin receptors in A549 cells

**Fig 3:**
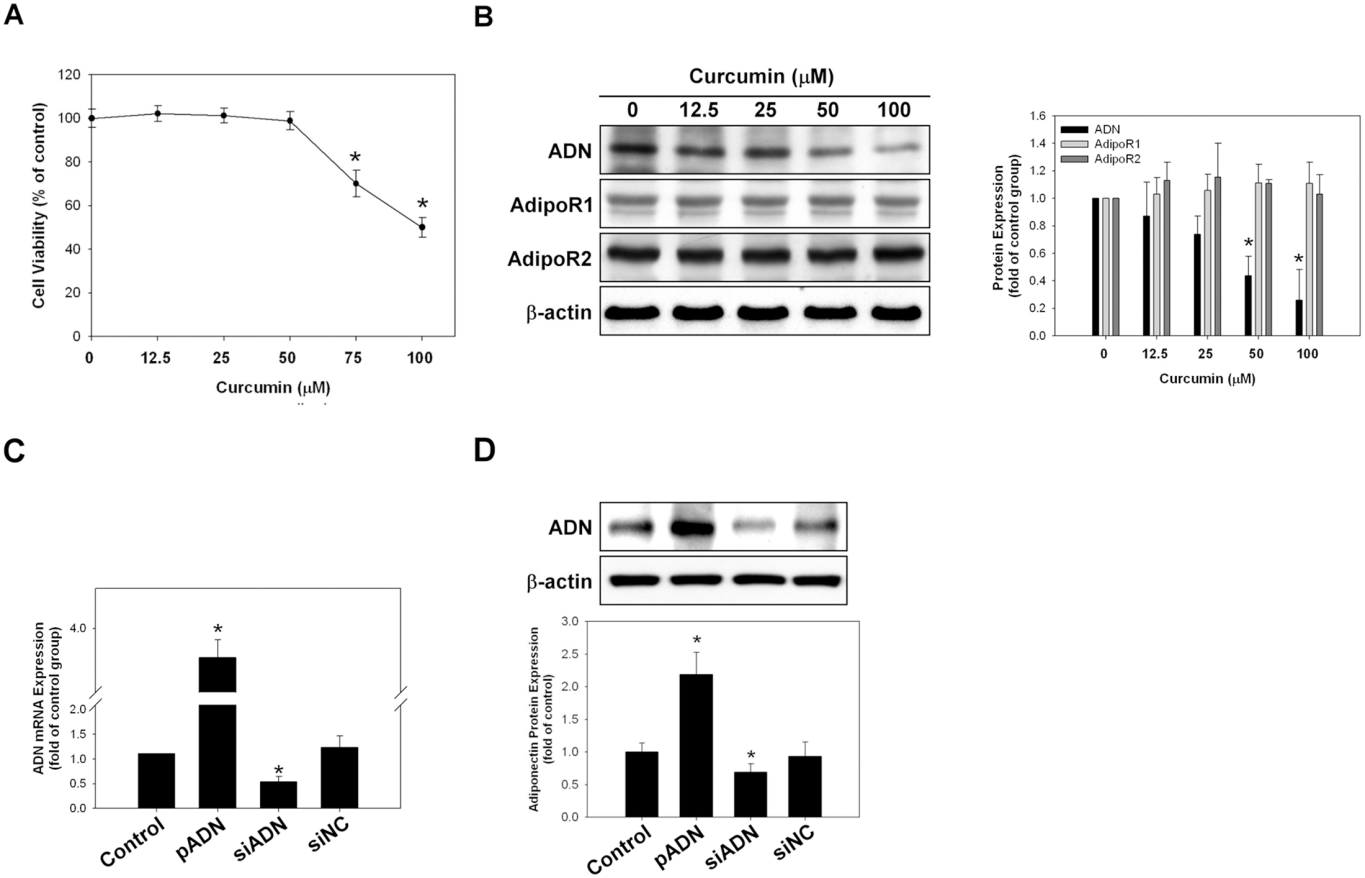
Effects of curcumin on cell cytotoxicity and on the expression levels of adiponectin, adiponectin receptor 1, and adiponectin receptor 2 in A549 cells. **(A)** Cell viability was analyzed by the MTT assay. The cytotoxic effect of curcumin was obvious at concentrations >75 μM. **(B)** Expression levels of adiponectin, adiponectin receptor 1, and adiponectin receptor 2 in A549 cells were analyzed by western blotting at different curcumin concentrations. **(C–D)** Exogenous induction and silencing of adiponectin expression in A549 cells. Adiponectin mRNA/protein expression in A549 cells increased significantly after exogenous adiponectin expression as analyzed by real-time PCR. Adiponectin expression decreased significantly after siRNA-mediated adiponectin silencing. NC-siRNA served as a negative control. Representative photos of three independent experiments. *p < 0.05 vs. control. The assays were conducted in triplicate.

The A549 cells were treated with different concentrations of curcumin. The MTT assay revealed that curcumin had a cytotoxic effect on the A549 cell lines at concentrations >75 μM (Fig 3A). Western blotting also revealed that the expression of adiponectin in A549 cells decreased significantly at a curcumin concentration of >50 μM (Fig 3B). The protein expression of adiponectin receptor 1 and adiponectin receptor 2 did not change with curcumin treatment even at high concentrations. The transfection of A549 cells with adiponectin vector or small interfering RNA (siRNA) plasmid successfully increased or silenced the expression of adiponectin, as demonstrated by polymerase chain reaction (PCR) and western blotting (Fig 3C and 3D).

### Effects of curcumin and adiponectin on the migratory and invasive ability of A549 cells

**Fig 4:**
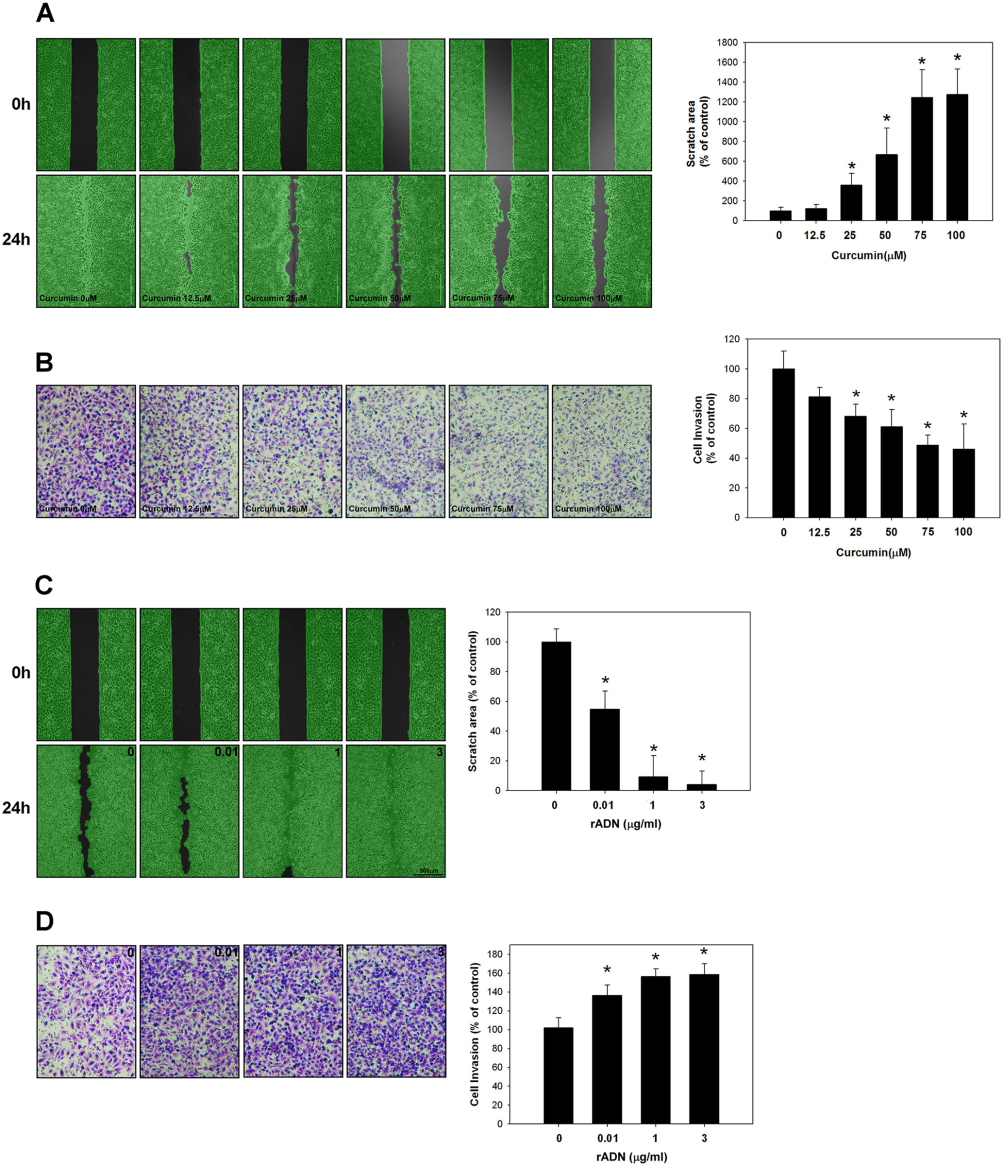
Effects of curcumin and recombinant adiponectin on the invasiveness/migration of A549 cells. **(A)** Cellular migration was determined using scratch wound assay and analyzed using the Wimasis WimScratch software. The migratory ability of A549 cells was significantly inhibited by increasing curcumin dosage compared with the control group (*p* < 0.05). **(B)** Invasiveness was determined by Matrigel-coated Boyden chamber assay. Curcumin significantly decreased the invasive ability of A549 cells (*p* < 0.05) when the curcumin concentration was >25 uM. **(C–D)** The application of recombinant adiponectin (rADN) significantly increased the migratory and invasive ability of A549 cells (*p* < 0.05). The results of three independent experiments are shown; the assays were conducted in triplicate.

Cellular migration was analyzed by wound scratch assay, while invasiveness was determined by Matrigel-coated Boyden chamber assay. Compared to the control group, the migratory and invasive ability of A549 cells was significantly inhibited with curcumin treatment in a dose-dependent manner (*p* < 0.05) (Fig 4A and 4B). On the other hand, recombinant adiponectin increased the invasive and migratory ability of A549 cells in a dose-dependent manner (*p* < 0.05) (Fig 4C and 4D).

### Regulatory roles of adiponectin receptor-1 and adiponectin receptor 2 in migration and invasiveness of A549 cells

**Fig 5:**
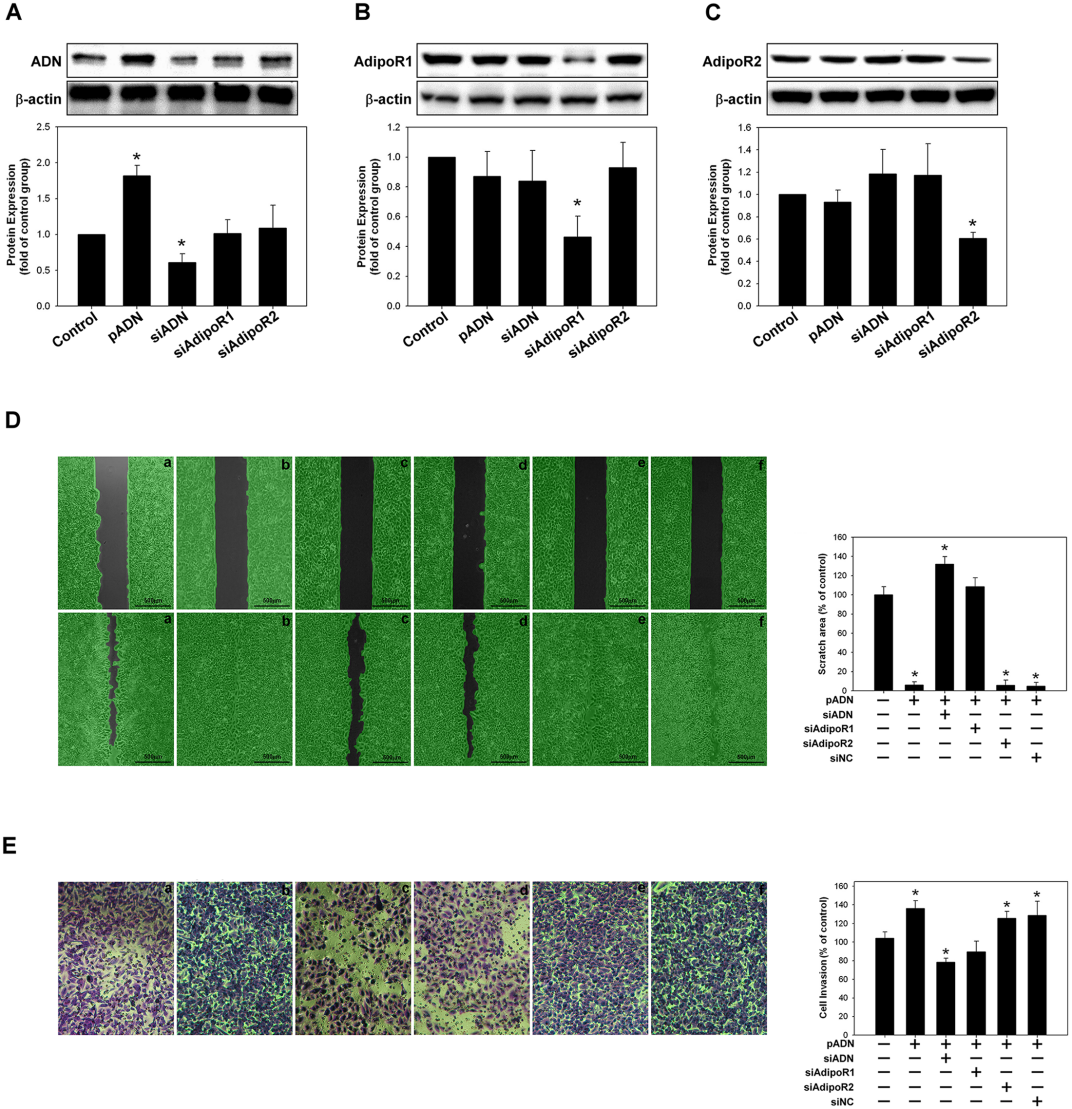
Adiponectin receptor 1 mediated the adiponectin effect on the migratory and invasive ability of A549 cells. **(A–C)** The expression levels of adiponectin, adiponectin receptor 1, and adiponectin receptor 2 were analyzed by western blotting after transfection with adiponectin vector or silencing by siADN, siAdipoR1, and siAdipoR2. **(D–E)** The migratory and invasive ability of A549 cells was inhibited after silencing of the adiponectin receptor 1 expression by transfection with siAdipoR1.

The A549 cells were transfected with adiponectin siRNA, adiponectin receptor 1 siRNA, and adiponectin receptor 2 siRNA plasmid. Plasmid transfection significantly reduced the expression levels of adiponectin, adiponectin receptor 1, and adiponectin receptor 2 in A549 cells, as confirmed by western blotting (Fig 5A–5C). Silencing the adiponectin expression did not affect the expression of both adiponectin receptor 1 and receptor 2, but silencing the expression of adiponectin receptor 1 blocked the effect of adiponectin, which enhanced the migratory and invasive ability of A549 cells. However, silencing the expression of adiponectin receptor 2 did not antagonize the effect of adiponectin on the migration and invasiveness of A549 cells (Fig 5D and 5E).

### Regulatory pathways of curcumin in adiponectin expression

**Fig 6:**
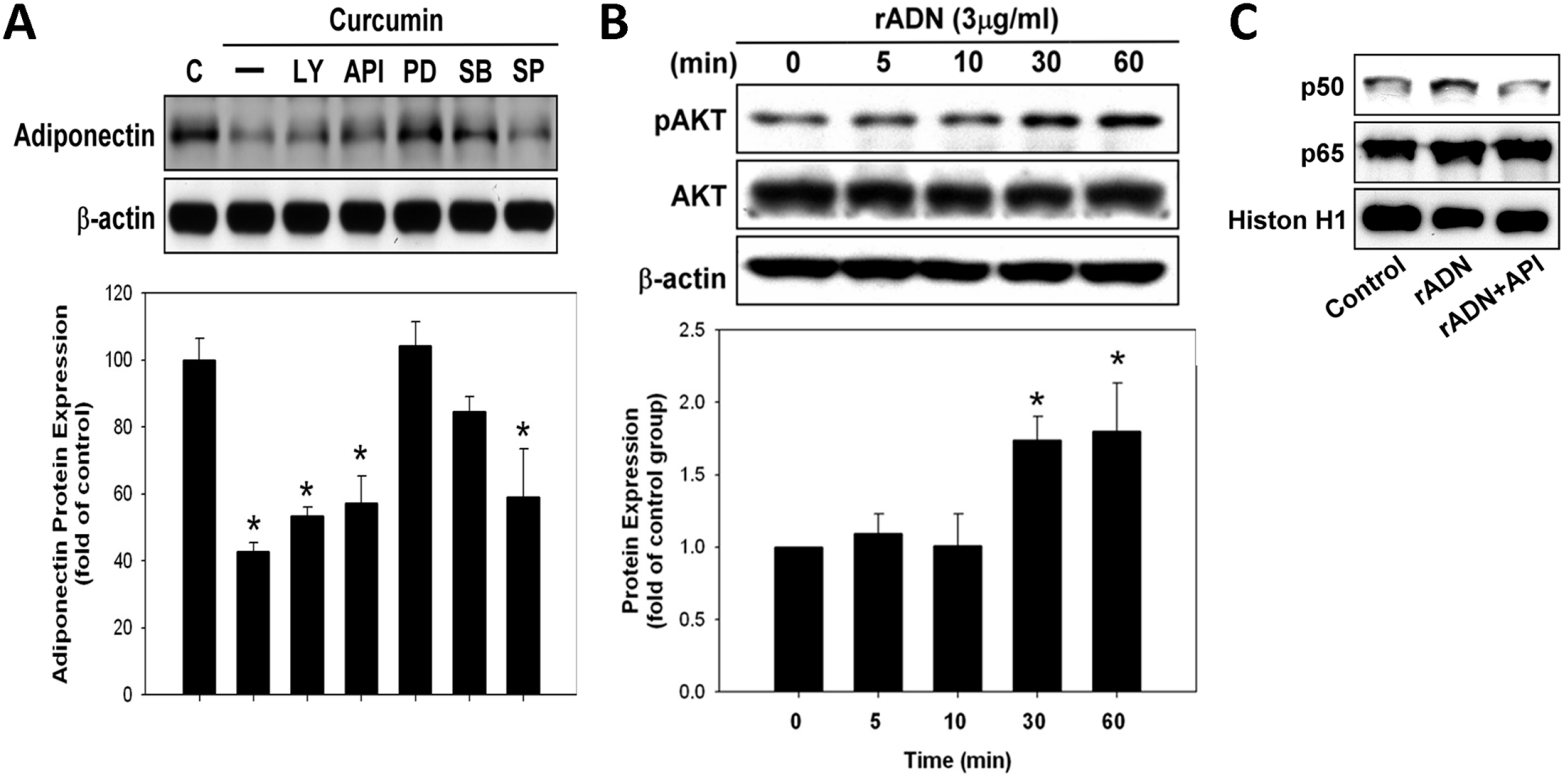
The regulatory pathways of curcumin on adiponectin expression. **(A)** A549 cells were treated with various PI3K/AKT and MAP kinase pathway inhibitors {(PI3K (LY294002; 10 μM), AKT (API-59; 3 μM), MAPK inhibitors [PD98059 (10 μM), SB203580 (10 μM), and SP600125 (10 μM) for ERK, p38–MAPK, and JNK, respectively]} for 1 h and later with curcumin (50 μM) for 24 h. Adiponectin expression was analyzed by western blotting. **(B)** AKT expression was analyzed by western blotting with different concentrations of recombination adiponectin. **(C)** The activity of p65/p50 of A549 cells was analyzed by EMSA after treatment with recombinant adiponectin or AKT inhibitor (API-59; 3 μM), respectively.

The A549 cells were treated with various types of inhibitors, including the inhibitors of PI3K/AKT and MAP kinase pathways {PI3K (LY294002; 10 μM), AKT (API-59; 3 μM), and MAPK inhibitors [PD98059 (10 μM), SB203580 (10 μM), and SP600125 (10 μM) for ERK, p38–MAPK, and JNK, respectively]}, for 1 h and later with curcumin (50 μM) for 24 h. The ERK and p38 pathway inhibitors blocked the inhibitory effect of curcumin on adiponectin expression (Fig 6A). Furthermore, recombinant adiponectin increased the expression of AKT (Fig 6B). Electrophoretic mobility shift assay (EMSA) showed that adiponectin regulated NF-κB expression through the AKT pathway (Fig 6C).

### Co-immuno-precipitation (Co-IP) assay

**Fig 7:**
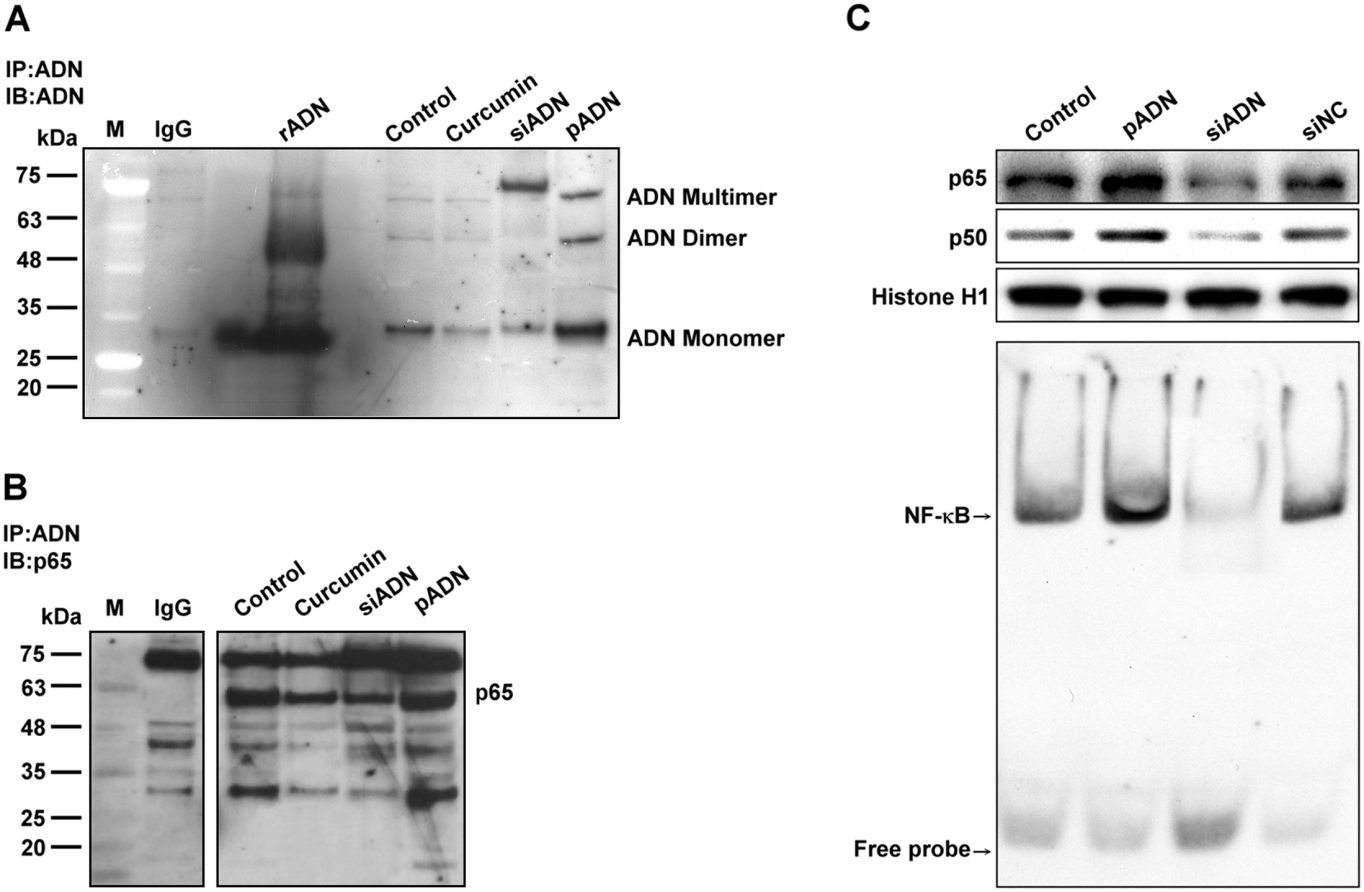
Co-immunoprecipitation assay of adiponectin interaction with p65. **(A)** A549 cells treated with curcumin, silenced, or transfected with adiponectin were lysed and adiponectin was co-immunoprecipitated using adiponectin antibody. Immunoblotting with the indicated antibodies confirmed the co-precipitation of adiponectin monomer, dimer, and multimer. **(B)** Co-immunoprecipitation was performed using anti-adiponectin and anti-p65 antibodies. The p65 elute was separated on SDS-PAGE and immunoblotted with the corresponding antibodies, which showed substantial association of adiponectin with the p65 component. **(C)** Activation of NF-κB by overexpression or silencing of adiponectin was determined by EMSA. Adiponectin increased the nuclear translocation of p65/p50 and NF-κB activation, but silencing adiponectin expression had the opposite effect.

This assay showed that adiponectin consisted of monomer, dimer, and multimer (Fig 7A). Curcumin and silencing adiponectin transfection successfully decreased the expression of adiponectin monomer and dimer. Curcumin also decreased the p65 expression, as shown by the Co-IP and EMSA assays (Fig 7B and 7C). Enhanced adiponectin expression by transfection with adiponectin also increased NF-κB expression. Adiponectin can translocate to nucleus to bind with nuclear p65.

### *In vivo* and *in vitro* effect of adiponectin on MMP expression

**Fig 8:**
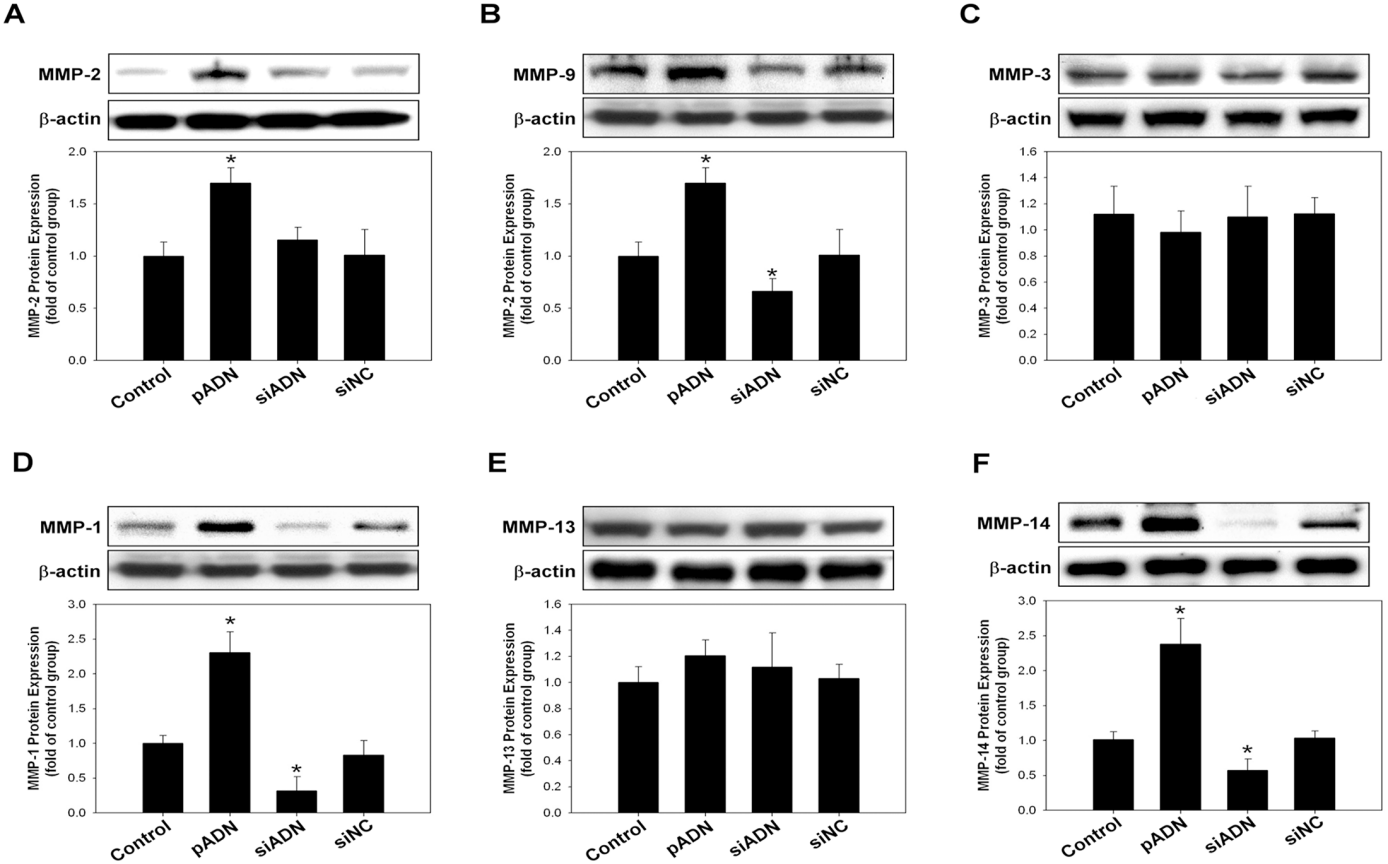
Overexpression or silencing of adiponectin expression on the matrix metalloproteinase (MMP) activity of A549 cells. Gelatin zymography was used to analyzed the activities of MMP-2/MMP-9, MMP-1/MMP-3, and MMP-13/MMP-14. **(A–F)** The activities of MMP-9, -1, and -14 were increased after the overexpression of adiponectin and decreased with the silencing of adiponectin. Expression of MMP-3 and -13 did not change regardless of adiponectin augmentation or silencing.

Transfection with adiponectin successfully increased the expression levels of MMP-2, -9, -1, and -14 in A549 cells. However, the expression of MMP-3 and -13 did not change with enhanced or silenced adiponectin expression (Fig 8).

### *In vivo* effect of curcumin on tumor growth and expression of adiponectin and MMPs

**Fig 9:**
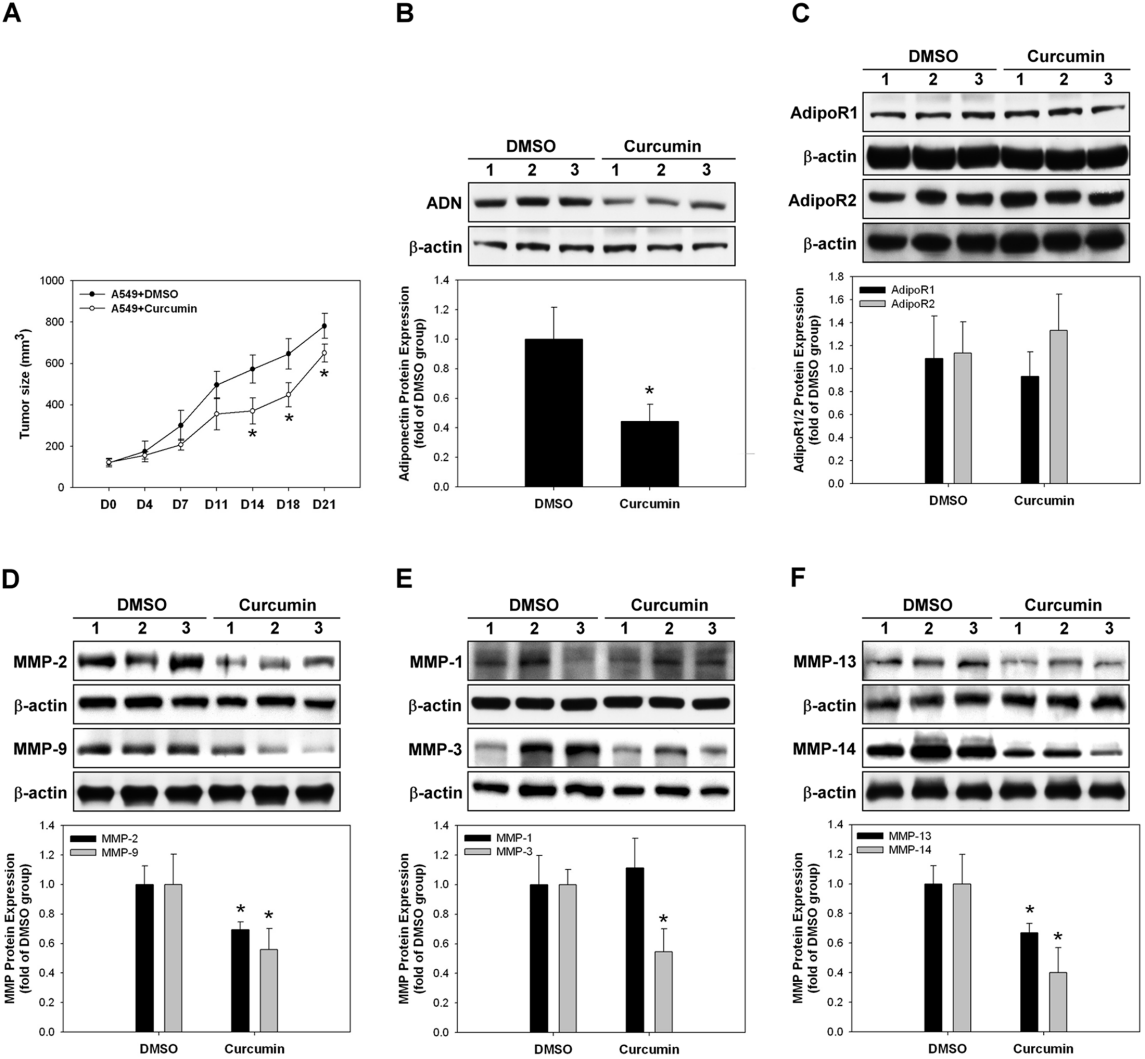
*In vivo* effect of curcumin on the expression of adiponectin and matrix metalloproteinases (MMPs). **(A)** The tumor sizes of curcumin-treated mice were decreased significantly compared to those of the control group after 14 days. **(B)** Tumor adiponectin expression of curcumin-treated mice was decreased significantly compared to that of the control group. **(C)** Expression of both adiponectin receptor 1 and receptor 2 in the curcumin-treated mice did not change *in vivo*. **(D–F)** Expression levels of MMP-2, -9, -3, -13, and -14 were decreased with curcumin treatment *in vivo*. MMP-1 expression was not altered.

Curcumin treatment significantly decreased the *in vivo* tumor growth and adiponectin expression compared with the control group (DMSO) (*p* < 0.05) (Fig 9A and 9B), but the expression of both adiponectin receptor 1 and receptor 2 did not change (Fig 9C). Curcumin treatment also significantly decreased the levels of all MMPs, except MMP-1 that remained uninfluenced, as compared with the control group (Fig 9D–9F).

## Discussion

Chemotherapy remains the primary treatment for advanced lung cancer[26], although targeted therapy is more popular. Serious side effects of chemotherapy usually limit their clinical application. Consequently, clinicians are becoming increasingly interested in herbal medicine since the past few decades because of its safety and effectiveness as an adjuvant therapy. Curcumin, a phenolic compound derived from the plant *Curcuma longa*, has been used for centuries for its anti-inflammatory, chemopreventive, and potential chemotherapeutic properties. Till date, current evidence shows that curcumin can block cancer cell proliferation, transformation, and invasion [27]. This study showed that curcumin can inhibit the migratory and invasive capacity of A549 cells *in vitro* in a dose-dependent manner. Moreover, curcumin also significantly decreased the *in vivo* tumor growth.

Tumor invasion and metastasis is a complicated multistep process that involves adherence, degradation of the surrounding matrix, migration, proliferation, and angiogenesis [28]. Curcumin possesses high natural antimetastatic ability. It can decrease the invasion of B16F-10 melanoma cells by inhibiting the expression of MMP-2 and MMP-9 and enhance the expression of antimetastatic proteins (E-cadherin and tissue inhibitors of MMP-2) [29,30]. In a xenograft tumor model of prostate or breast cancer, curcumin also showed high antimetastatic effect by suppressing the expression of MMP-9, NF-κB, and cyclooxygenase-2 [31,32]. In this study, curcumin can inhibit the migration and invasion of A549 in a dose-dependent manner *in vitro*. In our xenograft model with A549, curcumin can significantly decrease tumor growth and has a similar suppressing effect of the expression of MMPs, except for MMP-1 and -13.

Adipose tissue is considered as an endocrine system that can secrete a lot of hormones and cytokines, which are known as adipocytokines [33]. Adiponectin represents the most abundant adipose tissue protein with insulin-sensitizing, anti-inflammatory, and anti-atherogenic properties [34,35]. In addition, adiponectin may play a role in the development and progression of various types of malignancies [35]. The serum adiponectin level is inversely associated with the risk of obesity-related malignancies, including breast, endometrial, and prostate cancers, but it is positively correlated with gastric cancer and leukemia [35].

However, the prognostic role of circulatory adiponectin in lung cancer remains controversial [12,14]. In this report, adiponectin expression does not correlate to lung cancer TN and stages. Lung cancer patients with low adiponectin expression ratio showed longer survival time than those with high expression. Thus, adiponectin expression may be a prognostic factor of NSCLC.

In this study, we also demonstrated that NSCLC patients with metastasis have significantly higher adiponectin expression ratio than those without metastasis. The overexpression of adiponectin by transfection can increase the invasive and metastatic ability of A549 cells, perhaps through the activation of MMPs (1, 9, 14). Adiponectin can inhibit colon [36,37] and liver [37] cancer cell migration but promotes prostatic and endometrial carcinoma growth. Therefore, the role of adiponectin in carcinogenesis is variable in different cancer cell types.

Thus far, three types of adiponectin receptors have been identified: the two main receptors AdipoR1 and AdipoR2 and one receptor similar to the cadherin family. AdipoR1 and AdipoR2 are seven trans-membrane proteins that can mediate fatty acid oxidation and glucose uptake via adiponectin binding. Functional adiponectin receptors are normally expressed in lung epithelial cells. Using immunohistochemical staining, Jamshid et al. demonstrated that the expression of AdipoR1 and AdipoR2 is lower in NSCLC tissue than in normal lung tissue. Here, we demonstrated an increased AdipoR1 protein expression and immunohistochemical staining in NSCLC tissues. Silencing the AdipoR1 expression can block the effect of adiponectin on the migration and invasiveness of A549 cells. Blocking the AdipoR2 expression did not influence the migratory ability of A549 cells.

Curcumin has become an effective adjuvant agent in cancer therapy as it can modulate the activity of several transcription factors and their signaling pathways [38]. It can induce cellular apoptosis through the activation of the p38 pathway in lung cancer and ovarian tumor [39]. However, the regulatory role of curcumin in adiponectin expression remains unclear. We showed that curcumin inhibits adiponectin expression through the p38 and ERK pathways. Moreover, adiponectin can induce the activation of AKT pathway, which is also involved in the activation of NF-κB pathway in lung cancer.

In conclusion, adiponectin can be a novel potential prognostic marker of NSCLC. Except for classic chemotherapy, *in vivo* and *in vitro* results corroborate that curcumin may be used as an adjuvant agent in lung cancer therapy in the future. However, the poor absorption, metabolism, and bioavailability of curcumin may limit its clinical application. Several formulations have been prepared that include nanoparticles, liposomes, micelles, and phospholipid complexes, which could increase the bioavailability and promote longer circulation, better permeability, and resistance to metabolic processes of curcumin [40].

## Materials and Methods

### Tumor sample collection

The NSCLC and corresponding normal tissues were collected from 77 patients who underwent surgical resection between 2004 and 2011 at the Division of Thoracic Surgery, Department of Surgery, Kaohsiung Medical University Hospital. The tissue samples were immediately placed in liquid nitrogen for shipment to the laboratory and then stored in −80°C freezers until RNA isolation and protein extraction. Complete staging procedures, including chest radiography, bronchoscopy, computed tomography of the brain and thoracic cavity, sonography, and bone scintigraphy, were performed to precisely determine the tumor characteristics according to the TNM International Staging System for lung cancer [41]. All the patients were followed up until March 2012. Details of their demographic and survival data were updated.

### Ethics statement

The koahsiung medical university hospital’s Institutional Review Board for Research approved the study protocol (KMUH-IRB-990357). All of the participants provided written informed consent.

### RNA extraction and real-time PCR

Total RNA was isolated from frozen lung tumor tissues of NSCLC patients and corresponding normal adjacent lung tissues. RNA isolated from normal lung tissue, lung tumor tissue, and lung cancer cells was analyzed using real-time PCR, which was performed as described previously [42]. The following primers for real-time PCR were designed using the Primer Express software (RealQuant, Roche, Mannheim, Germany) using published sequences: human adiponectin sense primer: 5′-GGT GAT GGC AGA GAT GGC AC-3′ and adiponectin antisense primer: 5′- GCC TTG TCC TTC TTG AAG AG-3′ and human GAPDH sense primer: 5′-AGC CAC ATC GCT CAG ACA-3′ and GAPDH antisense primer: 5′-GCC CAA TAC GAC CAA ATC C-3′.

Fluorescence data were acquired at the end of the extension. Melt analysis was performed for all products to determine the specificity of the amplification. In addition, the PCR products were electrophoresed on 1% agarose gels to confirm the presence of correct band sizes. The relative expression was calculated as a ratio of the expression in the lung cancer tissue compared to the expression in the normal adjacent tissue (tumor lesion/normal tissue >1, high expression, and <1, low expression) [42–44].

### Immuno-histochemical staining

Tumor specimens were dissected from human lung tissue, fixed in 4% buffered formalin solution overnight, embedded in paraffin, and cut into 5-μm thickness sections. The paraffin sections were de-paraffinized with xylene and stained with anti-human adiponectin (GeneTex, Irvine, CA, USA), adiponectin receptor 1 (AdipoR1), and adiponectin receptor 2 (AdipoR2) (Santa Cruz, CA, USA). After washing with phosphate-buffered saline (PBS), the sections were incubated with horseradish peroxidase–conjugated secondary antibody for 1 h at room temperature. For color reactions, diaminobenzidine (DAB) was used and counterstained with hematoxylin. For negative controls, the antibody was replaced with control IgG.

### Western blotting

Western blotting was performed as described previously [42], after which the membranes were treated with PBS containing 0.05% Tween 20 and 2% skimmed milk for 1 h at room temperature and incubated separately with mouse anti-human adiponectin (GeneTex, Irvine, CA, USA), AdipoR1, AdipoR2, p65, p50, MMPs (2, 9, 1, 3, 13, 14) (Santa Cruz, CA, USA), Histone H1, AKT/pAKT, P38/pP38 (Cell Signaling, Danvers, MA, USA), and β-actin (Abcam, Cambridge, MA, USA) for 1 h. After washing, the membranes were incubated with horseradish peroxidase–conjugated rabbit anti-goat or mouse IgG at room temperature. Immunodetection was performed using chemiluminescence reagent plus NEN and exposure to Biomax MR Film (Kodak, Rochester, NY, USA).

### Culture of NSCLC cells

Lung adenocarcinoma A549 cells (ATCC CCL-185) were cultured in flasks containing F12K growth medium supplemented with 5% fetal bovine serum (FBS), 100 U/mL penicillin, and 100 pg/mL streptomycin at 37°C in a humidified atmosphere of 95% air and 5% CO_2_.

### Silencing the expression of adiponectin, adiponectin receptor 1, and adiponectin receptor 2 in A549 cells

siRNA, a specific double-stranded 21-nucleotide RNA sequence homologous to the target gene, was used to silence the expression of AdipoR1, and AdipoR2. Human adiponectin siRNA sense primer: 5′-GUG UGG GAU UGG AGA CUU ATT-3′ and antisense primer: 5′- UAA GUC UCC AAU CCC ACA CTT′-3 (Santa Cruz, CA); human AdipoR1 siRNA sense primer: 5′-GGA CAA CGA CUA UCU GCU ACA-3′ and antisense primer: 5′-UCU AGC AGA UAG UCG UUG UCC-3′; and AdipoR2 siRNA sense primer: 5′-GGA GUU UCG UUU CAU GAU CGG-3′ and antisense primer: 5′-CCG AUC AUG AAA CGA AAC UCC-3′ were used (Sigma-Aldrich, Singapore).

The silencing effect of siRNA transfection was assessed by real-time PCR and immunoblotting. Briefly, the cells were grown in a six well plate and transiently transfected with 20 nM siRNA using 8μL siPORT Amine (Ambion, Austin, TX, USA) for a total transfection volume of 0.5 mL. After incubation at 37°C, under 5% CO_2_ for 5 h, 1.5 mL of the normal growth medium was added, and the cells were incubated for 48 h.

### MTT assay for cell viability

MTT assays were performed as described previously [42].

### *In vitro* migration/invasion analyses

The migratory ability of the cells was assayed in a monolayer denudation assay, as described previously [42]. The confluent cells were wounded by scraping with a 100-μL pipette tip, which denuded a strip of the monolayer that was 300 μm in diameter. We washed the culture twice with PBS and added 5% FBS as a medium. After 24 hours, cells will migrated into the denuded area and were photographed. The areas were analyzed using the Wimasis WimScratch software, a new generation web-based image tool for cell migration analysis. Edge detection techniques can easily recognize the leading edge and the gap area. Users can upload the images and analysis will start automatically.

Cellular invasion was quantified using a modified Matrigel Boyden chamber assay. The BioCoat Matrigel invasion chamber (BD Biosciences, Bedford, MA) was used according to the manufacturer’s instructions. Cells (4 × 10^4^) in serum-free media were seeded onto Matrigel-coated filters. In the lower chambers, 5% FBS was added as a chemoattractant. After incubation for 24 h, the membrane was washed briefly with PBS and fixed with 4% paraformaldehyde. The upper side of the membrane was wiped gently with a cotton ball. The membrane was then stained using hematoxylin and removed. The magnitude of cell migration was evaluated by counting the migrated cells in 6 random clones under high power (×100) microscopic fields.

### Co-immunoprecipitation assay

To determine the protein–protein interactions between adiponectin and p65 (NF-κB subunit), the cells were harvested using immunoprecipitation lysis buffer (GeneTex). Nuclear proteins (800 μg) were cleared by incubating with protein A/G agarose beads (30 μL/tube), under rotating conditions, for 1 h at 4°C and then washed. The supernatants were collected and incubated with 2 μg of anti-adiponectin, anti-p65, or control rabbit IgG antibodies for 4 h. Protein A/G agarose beads (50 μL/tube) were then added to each tube, followed by overnight incubation at 4°C. The supernatants were removed by centrifuging at 12,000 *×g* for 10 min and disrupted by boiling in 1% SDS. The immune complexes were analyzed by immunoblotting with anti-adiponectin and anti-p65 antibodies.

### Mouse model

The animal use protocol was reviewed and approved by the Institutional Animal Care and Use Committee (IACUC) of Kaohsiung Medical University (IACUC 99067). Eight-week-old BC-17 SCID mice were purchased from Lasco, Taiwan. The effect of curcumin in attenuating NSCLC cell (A549) growth was assessed using a subcutaneous (s.c.) xenograft tumor model. Mice were subcutaneously inoculated in the right flank with 5 × 10^7^/ml cells, with one tumor site per rat. When the tumor growth reached 100 mm^3^, the mice received intraperitoneal injection (IP) of curcumin (45 mg/kg) or vehicle (DMSO) five times each week.

After two weeks of treatment with the above-mentioned agents, the tumors were isolated and the proteins were harvested and stored at −80°C. Some tumor tissues were collected in a tube containing 4% paraformaldehyde/PBS and stored at 4°C for 12 h. After washing three times with PBS, the tissues were embedded in paraffin and cut into 5-μm sections. Hematoxylin and eosin (H&E) and immunohistochemical staining were performed to examine the morphology and expression of adiponectin and MMP proteins in tumor tissues.

### Drugs and inhibitors

The PI3K/AKT and MAP kinase pathway inhibitors {PI3K (LY294002; 10 μM), AKT (API-59; 3 μM), MAPK inhibitors [PD98059 (10 μM), SB203580 (10 μM), and SP600125 (10 μM) for ERK, p38-MAPK, and JNK, respectively]}, and human adiponectin recombinant protein (Abcam, Cambridge, MA, USA) were used.

### Nuclear extract preparation and EMSA

Nuclear protein extracts were prepared as previously described and analyzed using EMSA [42,45].

### Statistical analyses

Statistical differences in the expression ratio of adiponectin and clinical parameters were tested using the student’s *t*-test or one-way analysis of variance (ANOVA). Survival curves were drawn using the Kaplan–Meier analysis. The results were expressed as mean ± SEM. Data were analyzed using the JMP software (SAS, JMP, Version 8.0, Cary, NC). The chi-square test was used for statistical analysis. One-way ANOVA was used to assess the differences among the groups. Statistical significance was set at *p* < 0.05.
